# Phone and e-mail counselling are effective for weight management in an overweight working population: a randomized controlled trial

**DOI:** 10.1186/1471-2458-9-6

**Published:** 2009-01-09

**Authors:** Marieke F van Wier, Geertje AM Ariëns, J Caroline Dekkers, Ingrid JM Hendriksen, Tjabe Smid, Willem van Mechelen

**Affiliations:** 1 Department of Public and Occupational Health/EMGO Institute, VU University Medical Center, Van der Boechorststraat 7, 1081 BT Amsterdam, the Netherlands; 2 Body@Work, Research Center Physical Activity, Work and Health, TNO-VUmc, Van der Boechorststraat 7, 1081 BT Amsterdam, the Netherlands; 3 Municipal Health Service The Hague, PO Box 12652, 2500 DP The Hague, the Netherlands; 4 TNO Quality of Life, Wassenaarseweg 56, 2333 AL Leiden, the Netherlands; 5 KLM Health Services, PO Box 7700, 1117 ZL Schiphol Airport, the Netherlands

## Abstract

**Background:**

The work setting provides an opportunity to introduce overweight (i.e., Body Mass Index ≥ 25 kg/m^2^) adults to a weight management programme, but new approaches are needed in this setting. The main purpose of this study was to investigate the effectiveness of lifestyle counselling by phone or e-mail on body weight, in an overweight working population. Secondary purposes were to establish effects on waist circumference and lifestyle behaviours, and to assess which communication method is the most effective.

**Methods:**

A randomized controlled trial with three treatments: intervention materials with phone counselling (phone group); a web-based intervention with e-mail counselling (internet group); and usual care, i.e. lifestyle brochures (control group). The interventions used lifestyle modification and lasted a maximum of six months. Subjects were 1386 employees, recruited from seven companies (67% male; mean age 43 (SD 8.6) y; mean BMI 29.6 (SD 3.5) kg/m^2^). Body weight was measured by research personnel and by questionnaire. Secondary outcomes fat, fruit and vegetable intake, physical activity and waist circumference were assessed by questionnaire. Measurements were done at baseline and after six months. Missing body weight was multiply imputed.

**Results:**

Body weight reduced 1.5 kg (95% CI -2.2;-0.8, p < 0.001) in the phone group and 0.6 kg (95% CI -1.3; -0.01, p = 0.045) in the internet group, compared with controls. In completers analyses, weight and waist circumference in the phone group were reduced with 1.6 kg (95% CI -2.2;-1.0, p < 0.001) and 1.9 cm (95% CI -2.7;-1.0, p < 0.001) respectively, fat intake decreased with 1 fatpoint (1 to 4 grams)/day (95% CI -1.7;-0.2, p = 0.01) and physical activity increased with 866 METminutes/week (95% CI 203;1530, p = 0.01), compared with controls. The internet intervention resulted in a weight loss of 1.1 kg (95% CI -1.7;-0.5, p < 0.001) and a reduction in waist circumference of 1.2 cm (95% CI -2.1;-0.4, p = 0.01), in comparison with usual care. The phone group appeared to have more and larger changes than the internet group, but comparisons revealed no significant differences.

**Conclusion:**

Lifestyle counselling by phone and e-mail is effective for weight management in overweight employees and shows potential for use in the work setting.

**Trial registration:**

ISCRTN04265725.

## Background

Globally more than one billion adults are overweight (i.e., having a Body Mass Index (BMI) > 25 kg/m^2^) and the numbers are still rising [1]. In the Netherlands nearly half of the adult population is overweight [2]. For those who are overweight, weight management (i.e., weight loss and/or prevention of weight gain) is important to alleviate overweight related health problems and to reduce chances of developing cardiovascular diseases and diabetes [3].

Few people appear to make use of professional help for weight management [4]. The reasons for this sparse use are not known, but clinicians not referring to professional help [5,6], financial costs, lack of time and personal preferences [7] could play a role. The work setting provides an opportunity to introduce a large group of adults to a weight management programme. Worksite interventions so far used various combinations of activities and the optimal design is not clear [8].

Weight loss programmes in the health care setting usually rely on lifestyle modification to change dietary intake and physical activity [9]. These strategies are known to produce weight loss [10,11]. Typically lifestyle modification is supported by (individual or group) face-to-face counselling, requiring multiple visits to a treatment facility. This may be less appealing to working adults, who are often constrained by lack of time for such programmes. Behaviour counselling by phone and e-mail (i.e., distance counselling) could be more feasible in the work setting. In other settings distance counselling has been applied to weight loss, dietary behaviours and physical activity. Phone counselling trials for weight loss, including trials primarily aimed at changes in diet and/or physical activity, showed mixed results [12-17]. The majority of phone counselling studies for physical activity and dietary behaviour found behaviour changes [18]. Few trials have investigated e-mail counselling for weight control or lifestyle behaviours. Those that did, found positive effects on body weight, mixed effects on diet [19,20] and no effect on physical activity [19-21]. Only one study recruited participants from a work setting [19]. We found no studies that directly compared the impact of phone counselling with e-mail counselling.

The main purpose of this study was to ascertain effects on body weight of a lifestyle programme with 10 biweekly counselling sessions by phone as well as by e-mail compared to self help materials, in overweight workers, at six months. Secondary purposes were to determine effects on waist circumference, diet and physical activity and to compare the effects of counselling by phone with the effects of counselling by e-mail.

## Methods

### Study design

The study was a three arm randomized controlled trial in which two arms received a six month lifestyle intervention with behaviour counselling by either phone (phone group) or e-mail (internet group). The third arm received usual care in the form of lifestyle brochures (control group). Details of the study design have been published elsewhere [22].

The study design, procedures and informed consent procedure were approved by the Medical Ethics Committee of the VU University Medical Center and all participants provided written informed consent.

### Participants

The logistics of the study dictated certain requirements of companies, like having a minimum of 1000 employees at one location or at close-by locations and the possibility to accommodate measurements at the worksite. The Human Resource Department and/or Occupational Health Department of potentially eligible companies were approached through four large occupational health services and through professional networks (e.g., the Netherlands Society of Occupational Medicine). Seven companies, i.e., two IT-companies, two hospitals, an insurance company, the head office of a bank and a police force, agreed to take part in the study. Over a period of six months approximately 21 000 employees were approached. In the insurance company employees were approached through a health fair and the company intranet. In the other companies all employees received a personal letter informing them about a lifestyle trial that was going to be carried out at their workplace and a screening questionnaire containing questions about the eligibility criteria. Around 25% of the employees was expected to meet the following criteria: BMI ≥ 25 kg/m^2^, paid employment for at least eight hours a week, able to read and write Dutch, having access to internet (either at work or at home) and skilled in using it, age at least 18 years, not pregnant and no diagnosis or treatment for disorders that would make physical activity difficult. Eligible employees received further study information and were invited to take part. If they affirmed the invitation, a personal appointment for the baseline body height and body weight measurements was made. BMI was calculated from these measurements; employees with a BMI < 25 kg/m^2 ^were subsequently excluded. Employees were then randomly assigned to one of the three study groups using a concealed allocation schedule based on permuted blocks to ensure equal distribution over the study groups in each company [22]. The participants were, in consequence of the nature of the intervention, not blinded for allocation after randomisation. They were not allowed to change groups.

An a priori power calculation to detect a weight loss of 1.4 kg (SD 6.8 kg) with 90% power in two-tailed tests at a significance level of 0.05, determined the sample size for the study at 1500 [22]. Loss to follow-up was not taken into account.

### Interventions

All groups received self-help materials published by the Netherlands Heart Foundation, intended for the general public. These materials dealt with overweight, healthy diet and physical activity. Additionally, the phone and internet group received a lifestyle intervention programme, which was adapted from previous work by HealthPartners in Minnesota, USA [16]. Based on principles of behaviour therapy [9], it consisted of ten modules. These modules provided information on nutrition and physical activity, and taught lifestyle modification strategies (e.g., self-monitoring, goal setting). Homework in the modules guided the participant in applying these techniques. Physical activity that employees could fit in their daily life (e.g., active commuting, walk at lunch) was encouraged. Participants received a pedometer (WA101, Oregon Scientific, Portland USA) to monitor their physical activity. Nutritional information stressed the reduction of calories by eating a healthy diet with less fat, sugar and alcohol. On the whole, the programme emphasized sustainable lifestyle changes rather than weight loss. After finishing each module, participants were contacted by their personal counsellor, depending on group allocation either by phone or by e-mail. Counselling was done by four trained counsellors (2 dieticians, two movement scientists) and according to two comparable standardized counselling protocols, one for each communication method [9,22]. Two weeks after randomisation, the counsellor initiated the intervention by contacting the employees. Participants could also contact the counsellor centre themselves.

#### Phone group

The phone group received the programme in a binder. Counselling sessions took place every two weeks, by appointment. In between contacts, the employee studied the module and completed the homework. This interactive process continued until the employee completed all modules, or until the participant declined contact.

#### Internet group

The internet group had access to an interactive website through a personal access code. Individualized web pages were generated from an underlying database containing general information and from the data that the participant entered in the modules. The counsellor was alerted when the employee finished a module, then checked the homework and commented on it through e-mail within five working days. When an employee did not log on to the website according to schedule, he/she received an e-mail reminder twice a week. Participants could also choose to be reminded by text messages on their mobile phone.

#### Control group

The control group received only the self-help materials and no counselling. At baseline the materials were briefly explained to the employee by the research personnel.

### Outcome measures

Outcomes of the study were change in body weight, waist circumference, dietary intake and physical activity between baseline and follow-up. Baseline and six month follow-up weight and height measurements were done at or near the workplace. No-shows were directly reminded by telephone and every effort was made to ensure that the weight measurement could be carried out. Self-reported outcomes were assessed at baseline and six month follow-up by a questionnaire which was sent to the home address of the participant. A maximum of five efforts over the course of two months was made to remind non-responders by mail, e-mail and phone.

Trained research personnel measured body weight and height according to measurement protocol [22]. Body weight (kg) was measured using a digital scale (Seca 770; Seca GmbH & Co, Hamburg, Germany), with participants wearing light clothing and no shoes. Besides the measured body weight, self-reported body weight was collected by questionnaire. If measured weight at follow-up was not present, but self-reported weight at baseline and follow-up was available, this was used in the analyses. In a separate study we found self-reported weight at baseline to be underreported by 1.4 kg [23], but we assume underreporting to be independent of time of measurement (i.e., baseline, follow-up) and group allocation. Relevant weight loss was defined as a decrease of at least 5% of initial weight as this is considered to be clinically relevant in obese individuals [24]. Weight maintenance was defined as avoiding a 3% increase in initial weight, as recently proposed [25]. Body height (cm) was measured at baseline with a portable stadiometer (Seca 214; Seca GmbH & Co, Hamburg, Germany). BMI was calculated by dividing the body weight (kg) by the square of body height (m^2^). Self reported waist circumference was measured with a non-tearing paper tape developed for the study [22,23].

The focus of dietary intake was on fat, fruit and vegetable intake in the previous month. Fat intake was assessed by the validated Dutch Fat List [26]. A total fat score was calculated (range 0 to 95), with one fat point representing a daily fat intake of between one and four grams of fat, two fat points representing five to eight grams of fat, et cetera. Vegetable intake in grams per day and fruit intake in pieces per day were determined from a validated short fruit and vegetable questionnaire [27,28]. For adults a daily intake of at least 200 grams of vegetables and two pieces of fruit is regarded to contribute to weight management [29].

Physical activity in the previous week was measured with the validated Short Questionnaire to Asses Health enhancing physical activity (SQUASH) [30]. This questionnaire inquires about duration (minutes), frequency (days per week) and perceived effort (light, average or heavy) spent on eight predefined activities and a maximum of four sports. MET values (multiplications of basic metabolic rate) were assigned to each activity and effort level, based on the compendium of activities developed by Ainsworth et al. [31,32]. Assigned MET values can be found in Additional file 1. MET-minutes per week were calculated for total physical activity. Adherence to the guideline of accumulating a minimum of 30 minutes of moderate physical activity on at least five days a week was assessed with a single question, asking about the number of days on which the respondent did at least 30 minutes of bicycling, gardening, odd jobs and sports.

Possible confounders and effect modifiers were measured by questionnaire. These included age, sex, educational level, country of birth, marital status, smoking behaviour, medication for certain health conditions and the number of previous weight loss attempts [22].

Lastly, counsellors tracked the content and number of counselling contacts in a web-based participant management system.

### Statistical analysis

Analyses to determine effectiveness were performed using multiple linear and logistic regression, with the follow-up outcome measure as the dependent variable. Assumptions of linear and logistic regression were verified. All analyses were adjusted for baseline values, thus creating an adjusted follow-up score [33]. Differences in effectiveness between counselling by phone and e-mail were assessed. For this two dummy variables were constructed and a simultaneous comparison with the control group was performed. Coefficients and confidence intervals in the phone group and the internet group were thereafter compared. If the confidence interval of the phone group included the coefficient of the internet group and/or vice versa, there was no significant difference.

All subjects, regardless of intervention adherence, were included in the analyses except respondents that became pregnant during the study. For the primary analysis on body weight, missing follow-up body weight was imputed. Body weight was considered missing if no follow-up weight measurement was performed and if self-reported body weight for both baseline and follow-up were unavailable. Five different data sets were created by applying multiple imputation using correlated variables such as baseline body weight, available body weight data from later follow-up measurements at 12 (self-reported), 18 (self-reported) and 24 months (measured or self-reported), age, sex and educational level in the imputation model [34]. These data sets were analysed as specified above. The estimates were then pooled with methods described by Rubin [35]. Secondary analyses were performed on complete cases for body weight, waist circumference, diet and physical activity.

In the secondary analyses on body weight, confounding was checked by adding a possible confounder to the regression model. A variable was classified as a confounder if the coefficient of group allocation had changed by 10%, compared to the coefficient of group allocation in the model without the variable. To examine effect modification, interaction terms were constructed and added to the regression model. If there were significant interaction effects, groups were stratified according to the identified effect modifier.

The multiply imputed datasets were generated using R version 2.7.1 [36]. Inferences from the primary analysis were pooled using Excel 2003. All analyses were performed with SPSS version 15.0 and p-values < 0.05 were considered significant.

## Results

### Participants

**Figure 1:**
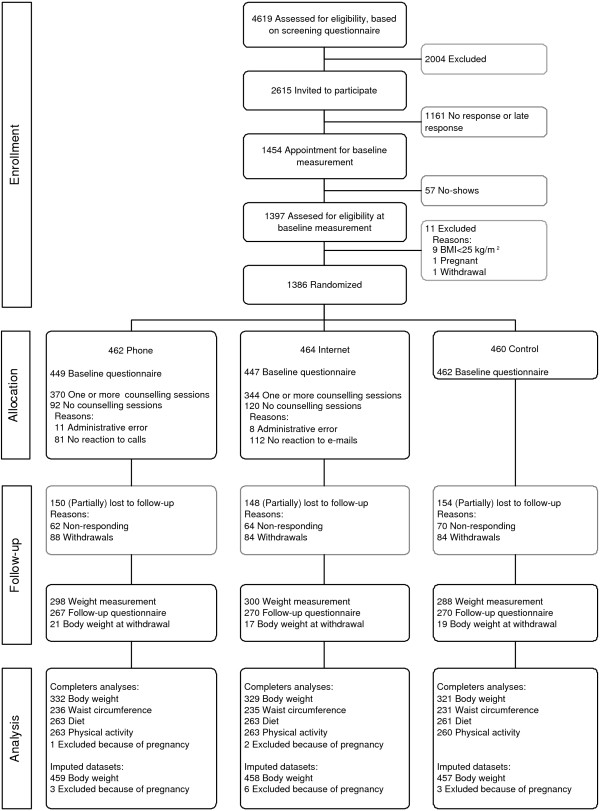
**Participant flowchart**. This chart illustrates the flow of participants through the trial and the response to the measurements. Analyses were performed for participants with either complete objective or complete subjective baseline- and follow-up data. Therefore the number of participants that was analysed in the completers-analyses is smaller than the number that responded to the follow-up measurements.

The screening questionnaire was returned by 4619 employees. Of these, 2615 were eligible to take part in the study. 1454 Employees were willing to participate and received an appointment for baseline measurement, which was kept by 1397 employees. At baseline 11 employees were excluded and 1386 employees were randomised to the phone group (N = 462), internet group (N = 464) and control group (N = 460). Participation in the study as a percentage of estimated number of eligible employees varied between 20% and 32% per company. The participant flow is presented in Figure 1.

Between baseline and one month after scheduled follow-up 256 participants withdrew from the study. Self-reported body weight at time of withdrawal was obtained from 57 participants. Most employees withdrew because of lack of time or motivation for further participation in the study and/or programme (30%), or because of personal and undisclosed reasons (34%). Nine participants withdrew because of pregnancy. These and three other pregnant participants were excluded from the analyses. Withdrawal was similar in the three groups. For 886/1386 (64%) participants measured body weight at follow-up was available. For 96/1386 (7%) directly measured follow-up weight was missing, but self reported weight at baseline and follow-up were present and subsequently used in the analysis. Data on participation in the intervention were available for all participants.

Baseline characteristics for participants are shown in Table 1. The majority was male (67%) and had a high education level (60%). Mean age was 43 (SD 8.6) years, mean BMI 29.6 (SD 3.5) kg/m^2 ^and 34% was obese. Approximately one out of six used medication for certain co-morbidities (hypertension (10%), hypercholesterolemia (6%), depression (3%), diabetes (2%), myocardial infarct (1%) and angina (1%)). Around two-third of the study population had previously tried to lose weight, of which 6.3% had used a formal weight loss programme. The majority wanted to lose weight.

**Table 1:** Baseline characteristics for all subjects, by treatment group

	**Phone** **n = 462**	**Internet** **n = 464**	**Control** **n = 460**	**All** **n = 1386**
Male, No. (%)	321 (69.5)	302 (65.1)	306 (66.5)	929 (67.0)

Age, mean (SD), y	43 (8.8)	43 (8.4)	43 (8.7)	43 (8.6)

BMI (SD), kg/m^2^	29.5 (3.5)	29.6 (3.4)	29.6 (3.7)	29.6 (3.5)

Highly educated, No. (%)^a^	271 (60.1)	281 (62.2)	255 (58.8)	807 (60.4)

Married/cohabiting, No. (%)^a^	380 (84.3)	384 (85.1)	368 (84.8)	1132 (84.7)

Born in the Netherlands, No. (%)^b^	416 (92.7)	417 (93.3)	401 (94.1)	1234 (93.3)

Medication for certain conditions, No. (%)^c^	80 (18.6)	77 (17.8)	67 (16.5)	224 (17.7)

Smokes ≥ 1 unit/day, No. (%)^c^	73 (16.3)	59 (13.2)	65 (15.3)	197 (14.9)

Weight loss attempts previous 2 yrs, No. (%)^e^				

0 attempts	147 (32.9)	141 (31.6)	141 (33.2)	429 (32.5)

1 – 3 attempts	212 (47.7)	202 (45.3)	196 (46.1)	610 (46.3)

4 or more attempts	88 (19.7)	103 (23.1)	88 (20.7)	279 (21.2)

Tried to prevent weight gain in previous 2 yrs, No. (%)^e^	353 (79.1)	370 (83.0)	347 (81.5)	1070 (81.2)

At baseline wants to, No. (%)^e^				

Loose weight	386 (86.2)	373 (83.8)	363 (85.6)	1122 (85.2)

Prevent weight gain	56 (12.5)	67 (15.1)	55 (13.0)	178 (13.5)

Neither are important	6 (1.3)	4 (1.6)	5 (1.1)	17 (1.3)

SD = Standard Deviation, ^a ^n = 1337, ^b ^n = 1322, ^c ^n = 1269, ^c ^n = 1320, ^e ^n = 1318

We compared participants with complete body weight data with the participants who had incomplete data. There was no differential non-response between groups in regard to numbers. However, employees with missing data had a higher baseline BMI (0.9 kg/m^2^, p < 0.001) and more were obese (40.6% vs. 31.5%, p < 0.001). Furthermore, they had a lower education level (53.6% high vs. 62.9 high, p < 0.001), contained more people that (tried to) quit smoking before or during the study (14.0% vs. 7.3%, p < 0.001), more people wanting to lose weight (89.7% vs. 83.4%, p = 0.01) and more people with 4 or more weight loss attempts (25.7% vs. 19.4%, p = 0.01) than the complete cases. A comparison of participants with complete lifestyle behaviour data with those with only baseline data showed equivalent differences. Additionally, employees with missing lifestyle follow-up consumed somewhat less vegetables (-9 gram/day, p = 0.029) and were less likely to eat two pieces of fruit per day (30.4% vs. 36.4%, p = 0.024).

### Participation in the intervention

**Figure 2:**
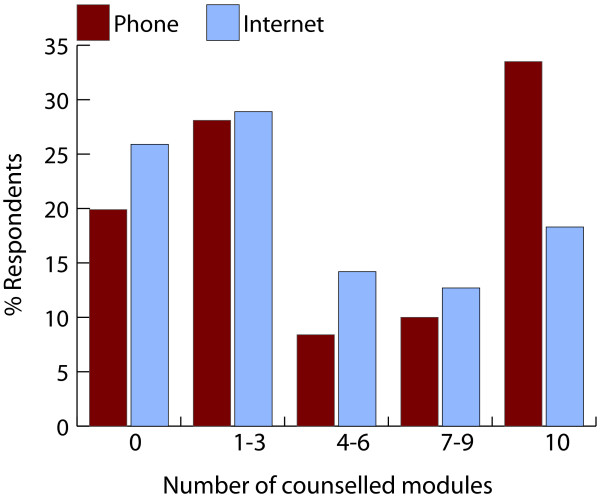
**Participation in the intervention**. The columns represent the proportions of participants in the phone and internet groups that did not receive any counselling (0) or that were counselled on 1–3, 4–6, 7–9 or 10 modules.

Erroneously 11 participants in the phone group and 8 participants in the internet group were never contacted by their counsellor. Of the participants in the phone group, 81 did not return the initiating calls from the counselling team, and 370/462 (80%) had at least one counselling session. The web-based programme was initiated by 400/464 (86%) employees and 344/464 (74%) employees were counselled on at least the first module. Figure 2 shows the attendance to the counselling sessions in each intervention group. The median number of counselled sessions for participants with complete body weight data was 9 (IQR = 2 to 10) modules in the phone group and 5 (IQR = 1 to 10) modules in the internet group. For participants with incomplete data this was 1 (IQR = 0 to 2) modules in the phone group and 0 (IQR = 0 to 2) modules in the internet group.

### Body weight

As table 2 shows, in the main analysis the phone group had a significant weight loss of 1.5 kg (95% CI -2.2; -0.8) in comparison with the control group. For the internet group this was 0.6 kg (95% CI -1.3; -0.01). The difference between the intervention groups was not statistically significant as their coefficients were mutually included in their 95% confidence intervals. The secondary analysis gave somewhat different results. In the phone group a significant loss of 1.6 kg (95% CI -2.2; -1.0) was established and in the internet group 1.1 kg (95% CI -1.7; -0.5), compared with the control group. No interaction or confounding was found.

**Table 2:** Baseline and follow-up anthropometric outcomes, by treatment group

	**Control group**		**Phone group**			**Internet group**		
	**Baseline**	**Follow-up**	**Baseline**	**Follow-up**	**Change vs. control (95% CI)**	**Baseline**	**Follow-up**	**Change vs. control (95% CI)**

**Imputed datasets**^a^	**n = 457**		**n = 459**			**n = 458**		

Body weight (SD), kg	92.9 (13.6)	91.7 (13.8)	93.4 (14.1)	90.7 (13.7)	-1.5*** (-2.2; -0.8)	92.8 (14.3)	91.0 (14.2)	-0.6* (-1.3; -0.01)

**Completers**	**n = 321**		**n = 332**			**n = 329**		

Body weight (SD), kg	92.0 (13.2)	91.0 (13.4)	93.5 (14.3)	90.8 (14.0)	-1.6*** (-2.2; -1.0)	91.9 (14.2)	89.8 (14.1)	-1.1*** (-1.7; -0.5)

≥5% weight loss, No. (%)	-	34 (10.6)	-	91 (27.4)	-	-	71 (21.6)	-

≥3% weight gain, No. (%)	-	26 (8.1)	-	20 (6.0)	-	-	18 (5.5)	-

	**n = 231**		**n = 236**			**n = 235**		

Waist circumference (SD), cm	101.5 (9.8)	99.5 (10.0)	102.6 (10.0)	98.6 (10.3)	-1.9*** (-2.7; -1.0)	101.5 (10.3)	98.2 (10.2)	-1.2** (-2.1; -0.4)

^a^All participants except those that became pregnant during the course of the study.* p < 0.05, ** p < 0.01, *** p < 0.001

Table 3 shows that in complete cases the phone and internet group were more likely to have a weight loss of at least 5% of initial weight than the control group. As presented in Table 2, in the control group the proportion achieving this result was 11%, in the phone and internet group it was 27% (OR 3.2 [95% CI 2.1; 4.9]) and 22% (OR 2.3 [95% CI 1.5; 3.6]) respectively. No significant results were found for likeliness to gain more than 3% of initial weight, with a proportion of 8% in the control group, 6% (OR 0.7 [95% CI 0.4; 1.3]) in the phone group and 5% (OR 0.7 [95% CI 0.4; 1.2]) in the internet group.

**Table 3:** Likeliness for meeting public health guidelines for weight control, waist circumference and lifestyle behaviours

	**OR (95% CI)**	**p value**
**Body weight loss ≥ 5%**		

Phone vs. Control	3.2* (2.1; 4.9)	<0.001

Internet vs. Control	2.3* (1.5; 3.6)	<0.001

**Body weight gain >3%**		

Phone vs. Control	0.7 (0.4; 1.3)	0.30

Internet vs. Control	0.7 (0.4; 1.2)	0.19

**≥ 200 gram vegetables/day**		

Phone vs. Control	1.0 (0.7; 1.7)	0.85

Internet vs. Control	0.9 (0.5; 1.4)	0.53

**≥ 2 pieces fruit/day**		

Phone vs. Control	1.1 (0.7; 1.6)	0.80

Internet vs. Control	0.9 (0.6; 1.4)	0.80

**≥ 30 mins. PA/5 days a week**		

Phone vs. Control	1.8* (1.3; 2.6)	<0.001

Internet vs. Control	1.4 (0.97; 2.1)	0.07

All analyses based on complete data. OR = odds ratio, PA = physical activity, *significant difference: p < 0.05.

### Waist circumference

Comparable results as for change in body weight were observed for reductions in waist circumference. Compared with the control group, the phone group significantly lost 1.9 cm (95% CI -2.7; -1.0) and the internet group 1.2 cm (95% CI -2.1; -0.4), as can be seen in table 2. No differences were found between counselling by phone and e-mail.

### Dietary behaviour and physical activity

Table 4 presents the behavioural outcomes. The comparison of the phone group with the control group showed statistically significant changes for fat intake and for physical activity. Fat intake decreased by 1.0 fat points (95% CI -1.7; -0.2), representing 1 to 4 grams per day, more in the phone group; no differences were seen between the phone and internet group. The phone group also showed a significant increase in physical activity by 866 METminutes (95% CI 203; 1530) and an odds ratio of 1.8 (95% CI 1.3; 2.6) for likeliness to adhere to the physical activity guideline (Table 3), compared with the control group, though no differences were found in the comparison of the phone group and the internet group.

**Table 4:** Baseline and follow-up lifestyle behaviour outcomes, by treatment group

	**Control group**		**Phone group**			**Internet group**		
	**Baseline**	**Follow-up**	**Baseline**	**Follow-up**	**Change vs. control (95% CI)**	**Baseline**	**Follow-up**	**Change vs. control (95% CI)**

**Diet**	**n = 261**		**n = 263**			**n = 263**		

Fat (SD), score/day	18.6 (6.2)	16.7 (5.9)	18.6 (5.9)	15.7 (5.5)	-1.0* (-1.7; -0.2)	18.0 (6.3)	15.6 (5.9)	-0.7 (-1.4; 0.04)

Vegetables^a ^(IQR), g/day	143 (100; 193)	143 (100; 193)	136 (93; 193)	143 (100; 193)	9 (-1; 20)	129 (86; 171)	129 (100; 186)	-2 (-12; 9)

≥ 200 g veg./day, No (%)	54 (20.7)	56 (21.5)	50 (19.0)	56 (21.3)	-	47 (17.9)	48 (18.3)	-

Fruit^a ^(IQR), pieces^b^/day	1.6 (1.0; 2.6)	1.9 (1.0; 2.9)	1.7 (0.9; 2.6)	2.0 (1.3; 2.6)	0.2 (-0.02; 0.4)	1.6 (0.9; 2.4)	1.7 (1.3; 2.4)	-0.04 (-0.2; 0.2)

≥ 2 pieces fruit/day, No (%)	96 (36.8)	109 (41.8)	100 (38.0)	114 (43.3)	-	90(34.2)	104 (39.5)	-

**Physical activity**	**n = 260**		**N = 263**			**n = 263**		

Total PA^a ^(IQR), METmins./wk.	6114 (3273; 8755)	5940 (3596; 9141)	5895 (3250; 8690)	6875 (4645; 9483)	866* (203; 1530)	6060 (3240; 8355)	7080 (4260; 9145)	431 (-233; 1095)

≥ 30 mins./5 days a week, No (%)	91 (35.0)	100 (38.5)	83 (31.6)	131 (49.8)	-	81 (30.8)	116 (44.1)	-

All analyses based on complete data.

^a^Median, ^b^One piece or portion of fruit approximates 100 grams.

IQR = interquartile range, PA = physical activity, * p < 0.05, ** p < 0.01, *** p < 0.001

## Discussion

Our study shows that a lifestyle programme combined with a maximum of 10 counselling sessions in six months, aimed at overweight workers, is effective for reducing body weight by 1.5 kg if counselling is done by phone and 0.6 kg if counselling is done by e-mail, compared to self help materials. Distance lifestyle counselling is also effective for producing clinically relevant weight loss. No effect was found for avoiding a 3% weight gain. The weight reduction from counselling by phone was higher than weight loss found at six months in two other studies [15,16]. Nevertheless, results in both intervention groups seem lower than those seen in other distance counselling studies [17,19,20]. An explanation for the larger effect on weight loss in the studies by Tate et al. [19,20] is their explicit recommendation of a maximum daily intake of 1500 kcal, while we focused on a healthy diet. Furthermore, these studies offered more frequent contact, ranging from daily to weekly phone calls or e-mails, than we did. Their effects are in line with results from a meta-analysis [11], showing that increasing the counselling intensity significantly increases weight reduction. Increasing intensity also raises the costs of a behavioural intervention programme. Future research should study the cost-effectiveness of different intensities.

We have also shown that the lifestyle programme with distance counselling is effective for reducing waist circumference by 1.9 cm in the phone group and 1.2 cm in the internet group, compared with self help materials. Tate et al. [19] found larger waist circumference reductions from e-mail counselling, but these reductions are probably associated with the higher weight loss that was produced in their study.

Phone counselling resulted in an intra-group reduction of 2.7 fat points, representing 6–8 grams of fat and a reduction of 54–72 kcals per day. In an average diet of 2250 kcals per day this would constitute a 2.4–3.2% reduction in energy from fat. Another study, emphasizing much lower fat intakes than we did, showed less reduction [17], while a second study showed a larger reduction in the intake of total fat than we achieved [15]. This study was performed in cardiac patients that were counselled to lower their blood cholesterol. Maybe they were more motivated to change fat-intake than our overweight subjects. Nonetheless, the effect we find on fat consumption in the phone group is substantial and constitutes a meaningful contribution to weight management.

We found no intervention effects on the consumption of vegetables or fruit at six months. With regard to vegetable consumption this could be explained by a ceiling effect. Mean intake at baseline was already close to the, in The Netherlands, recommended minimum intake of 150 g/day. Alternatively, in our programme fruit and vegetables were recommended as 'healthy' choices, but their importance for weight regulation was not discussed. Whether emphasizing the role of fruit and vegetables for weight control increases their consumption should be further studied.

Physical activity levels increased as a result of the intervention, but only the phone group showed a significant difference compared with the control group. This is in agreement with studies that found increased physical activity from phone counselling [37,38] and no effect from internet counselling [21].

Attendance to the counselling sessions was satisfactory in individuals with complete data, but low in those with missing data. Attendance has been found to be associated with weight loss [14,16,19], so improving attendance could increase weight loss. However, the question remains if attendance to counselling sessions is responsible for successful weight change, or rather if it is a representative of an underlying motivational construct that also influences behaviour change.

A secondary aim of the study was to determine differences in the effects of phone counselling and e-mail counselling. With regard to fat intake and physical activity, the phone group appears to perform better than the internet group, because only in this group significant changes in comparison with the control group were seen. In addition, changes in the phone group are larger than in the internet group but direct comparisons between the phone and internet group showed no statistical differences.

Several potential limitations in this study need to be considered. First, for 29% of the participants no follow-up data on body weight at six months were available. This is comparable to other distance counselling studies [16,17,19] and lower than in some studies in the work setting [39,40]. Missing data has implications. Results from completers-analyses and from analyses for which the baseline value is carried forward, are only valid if data are missing completely at random [41]. The comparison between completers and non-completers showed that missingness was associated with observed data like baseline body weight and counselled modules. We therefore based our imputation model on missing at random (MAR) assumptions and included all variables that were related to the variables with missingness in our imputation model. An advantage of multiple imputation over single imputation methods is that it allows for the uncertainty of the values that are used to substitute the missing values [41]. The results we found after multiple imputation differed from the completers-analyses, especially for the internet group, but are more credible because of the MAR assumption and the use of multiply imputed datasets.

A second restriction is that analyses of waist circumference and of the behavioural outcomes were limited to complete cases. Loss to follow-up was non-differential. However, in the intervention groups, participants that completed follow-up measurements had also completed more modules compared to the participants with missing follow-up. As argued before, attendance to the sessions could be indicative of adherence to behaviour change. Thus non-responders and dropouts in the intervention groups would have fewer or no change in their diet and physical activity behaviour than responders. Although non-responders and dropouts in the control group can be assumed to be equally (non)adherent to these behaviour changes, effects in all participants are probably attenuated compared to the complete-case-analysis.

A further consideration is whether the effects we found on body weight are meaningful. From the individual viewpoint additional weight loss of 1.5 kg or 0.6 kg (i.e., the mean weight losses in the phone and internet group compared to self-help materials) is not the amount wished for. However, as Rose has argued, small changes in a large group can have a huge impact on public health [42]. A modelling study showed that reducing BMI by 2 points in a moderate to high risk group (BMI ≥ 24) has considerable effect on the population burden of diabetes [43]. The type of programme we studied can be used to reach a large group of overweight employees; we managed to engage about 25% of the overweight working population. We therefore consider our results to be of relevance for public health. Further research should elicit if they are sustainable and cost-effective.

Other limitations of our study are that behavioural outcomes are all based on self-report and that we only measured a few of the dietary changes associated with weight control. We found that an exhaustive food questionnaire increased our questionnaire to unacceptable length. For that reason we focused on fat, fruit and vegetable intake. More objective measurement of lifestyle behaviours was not feasible because of the trial size. Self-report is vulnerable to social desirability bias which especially at follow-up might have led to more favourable outcomes.

Lastly, the study population does not represent the general Dutch working population (40% high educated, 57% men). This is related to the fact that we mostly included companies that employ white collar workers. Also, self-selection of more health oriented workers probably took place judged by baseline adherence to public health guidelines which is higher than found in the general population and by the proportion of smokers which was lower than expected on the basis of education level and age. This is a common phenomenon in lifestyle interventions, demonstrating that it is hard to engage those who, from the public health perspective, are most in need of change. When an intervention like ours is implemented in the work setting, efforts should be made to recruit lower-educated and high-risk individuals, and effects from the intervention in this population should be evaluated.

Strengths of our study include objective measurement of body weight, broad inclusion criteria, size of the group studied, use of multiple imputation for missing data, recruitment of individuals who previously had not been engaged in weight loss programmes and the design of an intervention suitable for the occupational setting. We are therefore confident that the programme we developed and the results we found are transferable to the occupational health practice.

## Conclusion

Results showed that lifestyle counselling by phone and e-mail is effective for reducing body weight and waist circumference in a group of overweight employees at six months. Furthermore, counselling by phone is effective for reducing fat intake and increasing physical activity.

## Competing interests

The authors declare that they have no competing interests.

## Authors' contributions

WM and GAMA conceived of the study. JCD and MFW acquired the data. MFW performed the statistical analysis and interpretation of data and drafted the manuscript, under supervision from WVM, GAMA and JCD. All authors participated in the design of the study, in the revision of the manuscript and read and approved the final manuscript.

## Pre-publication history

The pre-publication history for this paper can be accessed here:



## Supplementary Material

Additional file 1 **Assigned MET-values for the self rated effort levels in each activity domain.** Based on the compendium of activities developed by Ainsworth and colleagues [31,32]. Click here for file
