# Supplementary material for: Phone and e-mail counselling are effective for weight management in an overweight working population: a randomized controlled trial
Source: BMC Public Health. 2009 Jan 9;9:6. doi: 10.1186/1471-2458-9-6 (PMC2667416; doi:10.1186/1471-2458-9-6)
Supplement: Additional file 1 — Assigned MET-values for the self rated effort levels in each activity domain. Based on the compendium of activities developed by Ainsworth and colleagues [31,32]. [file 1471-2458-9-6-S1.doc]

| ***Activity*** | ***Effort level*** | ***MET-value*** | ***Ainsworth codes*** | ***Ainsworth descriptions*** |
| --- | --- | --- | --- | --- |
| Walking - to and from work  - leisure time (including leisure time transportation) | Light | 2.5 | 17152, 17161 | walking to and from transport, 3.2 km/h |
|  | Average | 3.5 | 17160, 17250 | walking for pleasure, work break |
|  | Heavy | 3.8 | 17200 | brisk pace, 5.6 km/h |
| Bicycling - to and from work  - leisure time (including leisure time transportation)  N.B. Average bicycling velocity is 18 km/h in the Netherlands | Light | 4.0 | 1010 | <16 km/h, leisure, to work or for pleasure |
|  | Average | 7.0 | 1020, 1030 | (mean value of) light (6.0 MET) to moderate effort (8.0 MET), 16 - 22 km/h |
|  | Heavy | 10.0 | 1040 | vigorous, 22-25.5 km/h |
| Gardening | Light | 2.5 | 8220 | walking, seeding lawn (watering the garden is 1.5 MET, picking up yard is 3.0 MET) |
|  | Average | 4.0 | 8245 | gardening general |
|  | Heavy | 5.5 | 8095 | mowing lawn general (highest MET-value in lawn and garden activities is 6.0) |
| Home repair | Light | 3.0 | 6040, 6160 | carpentry, general workshop; painting, papering |
|  | Average | 4.5 | 6130, 6190 | laying or removing carpet; sanding floors |
|  | Heavy | 6.0 | 6050, 6180 | carpentry outside house; roofing (the only other high MET-value is 7.5 for sawing hard wood) |

| ***Activity*** | ***Effort level*** | ***MET-value*** | ***Ainsworth codes*** | ***Ainsworth descriptions*** |
| --- | --- | --- | --- | --- |
| Occupational activities  N.B. The majority of respondents in our study were office workers | Light & average Examples: sitting, standing, walking, carrying light objects | 2.0 | 11580, 11610, 11526 | sitting, light office work (1.5 MET); standing, light moderate (including patient care) (3.0 MET); driving police squad car (2.0 MET) |
|  | **Heavy**  Examples: lifting or carrying heavy objects | 5.0 | 11820 | carrying objects of 11 to 22 kg while walking or standing |
| Home (household) activities | Light & average Examples: standing tasks like cooking and doing the dishes; walking tasks like vacuum cleaning and grocery shopping. | 3.0 | 5025, 5026 | (mean value of) multiple household tasks light (2.5 MET) and multiple household tasks moderate (3.5 MET) |
|  | **Heavy**  Examples: scrubbing floors, beating carpets, carrying heavy groceries | 4.5 | 5027 | multiple household tasks vigorous |
| Sports activities | Sports activities were coded depending on type of activity and indicated effort level matching descriptions in the compendium. Instructions for the coding rules can be obtained from the authors. | | | |
